# The Effects of Glyphosate and Roundup^®^ Herbicides on the Kidneys’ Cortex and the Medulla and on Renal Tubular Cells’ Mitochondrial Respiration and Oxidative Stress

**DOI:** 10.3390/molecules30112335

**Published:** 2025-05-27

**Authors:** Rayhana Rihani, Anne-Laure Charles, Walid Oulehri, Bernard Geny

**Affiliations:** 1 Biomedicine Research Center of Strasbourg (CRBS), UR 3072, “Mitochondria, Oxidative Stress and Muscle Plasticity”, Faculty of Medicine, University of Strasbourg, 67000 Strasbourg, France; rayhana.rihani@etu.unistra.fr (R.R.); anne.laure.charles@unistra.fr (A.-L.C.); walid.oulehri@chru-strasbourg.fr (W.O.); 2 Department of Anesthesiology, Faculty of Medicine, University of Strasbourg, 67000 Strasbourg, France; 3 Department of Physiology and Functional Explorations, Faculty of Medicine, University Hospital of Strasbourg, 67091 Strasbourg, France

**Keywords:** herbicide, glyphosate, Roundup^®^, kidney, renal cortex, medulla, HK2 cells, mitochondria, mitochondrial respiration, oxidative stress, hydrogen peroxide (H_2_O_2_)

## Abstract

Glyphosate (GP) and its derivatives are present in almost all environments and suspected to induce acute and chronic kidney injuries. This public health issue is relatively underexplored. We therefore conducted an investigation on rats and tubular HK2 cells cultured for 24 h to determine whether GP’s and Roundup’s^®^ (RU) potential renal toxicity might be related to mitochondrial respiration impairment and the increased production of hydrogen peroxide (H_2_O_2_) in both the renal cortex and medulla (involved in filtration and reabsorption, respectively) using a high-resolution oxygraph (Oxygraph-2K, Oroboros instruments). GP alone decreased maximal uncoupled mitochondrial respiration in the medulla (−14.2%, *p* = 0.02). RU decreased mitochondrial respiratory chain complexes I and I + II and the maximal respiratory capacity both in the renal cortex (−13.5%, *p* = 0.04; −20.1%, *p* = 0.009; and −14.7%, *p* = 0.08, respectively) and in the medulla for OXPHOS I + II (80.82 ± 7.88 vs. 61.03 ± 7.67 pmol/(s·mL), −24.5%, *p* = 0.003). Similarly, in HK2 cells, the decrease in OXPHOS CI + II was greater after RU (65.87 ± 1.30 vs. 51.82 ± 3.50 pmol/(s·mL), −21.3%, *p* = 0.04) compared to GP. Increased H_2_O_2_ production was mainly observed after RU in the medulla (+14.3% in OXPHOS CI + II, *p* = 0.04) and in HK2 cells (+19% in OXPHOS CI + II, *p* = 0.02). In conclusion, although the medulla might be more prone to GP-related mitochondrial damage, RU toxicity was greater in both the renal cortex and medulla and in cultured tubular HK2 cells. Enhancing mitochondrial respiration and reducing oxidative stress might favor the prevention of or reduction in such worldwide-used herbicides’ deleterious effects on the kidneys.

## 1. Introduction

Among potentially toxic substances, pesticides and herbicides are in first place since they are used worldwide and can induce deleterious effects in many systems and organs. Herbicides are used with the aim of protecting crops against weed infestation, but increasing evidence supports their deleterious effects on the environment, including water, soil, and air, and on humans, animals, and microorganisms. Thus, the poorly controlled use of herbicides likely results in adverse effects on non-target organisms [1].

More specifically, glyphosate and its derivatives are present in almost all natural and agricultural environments, as well as in plants and seeds. Thus, for instance, glyphosate is used in Amazonia on guarana. It causes damage whatever the dose, but lower doses do not interfere with seedling growth or development [2]. This herbicide is found in many parts of the plant, such as the leaves, stems, and spikelets of dried wheat. It has also been detected in white and brown rice, major nutriments for billions of people [3,4]. Glyphosate residues are observed in water all around the world, such as in European countries, Canada, the USA, Mexico, and Argentina, and, accordingly, it is found in drinking water [5,6,7]. As a consequence, consistent reports demonstrate its presence in animals and in the human body [8,9].

Similarly to the fact that glyphosate’s deleterious health effects are increased when combined with exposure to other pollutants [10], glyphosate formulation with surfactants appears to have worse effects when considering health. Glyphosate is generally not used alone because adding surfactants improves its efficacy. Although the exact composition of the co-formulants in glyphosate-based herbicides remains undisclosed to the public, polyethoxylated tallow amine surfactants are common in glyphosate preparation. Importantly, the potential toxicity of surfactants appears greater, and, for instance, Roundup’s^®^ formulation demonstrated enhanced toxicity compared to glyphosate alone [11,12,13,14].

Interestingly, relatively little data are available on the effects of glyphosate and Roundup^®^ on the kidneys despite the major physiological role of renal function in systemic homeostasis. Indeed, besides participating in blood pressure regulation, the kidneys are responsible for blood filtration and, ultimately, the elimination of waste. More specifically, the renal cortex contains almost all the glomeruli and is primarily involved in blood filtration. The renal medulla, composed of tubules, allows for the reabsorption of water, salt, and many substances, thus contributing to homeostasis. Well-functioning kidneys are therefore needed to support a healthy life, even though they are continuously in contact with potentially toxic compounds like herbicides.

Thus, after exposure, about 20 to 30% of glyphosate is eliminated in the urine, and the highest glyphosate concentration is observed in the kidneys compared to other tissues [8]. Recently, Lin Hu et al. observed an association between exposure to glyphosate and impaired renal function in adults [15]. The mechanisms involved are not totally understood, but environmentally relevant concentrations of Roundup^®^ disrupt kidney architecture, enhance oxidative stress, and induce cellular apoptosis [16]. Furthermore, glyphosate ingestion can result in acute tubulointerstitial damage involving abnormal mitochondria [17,18]. Besides tubular lesions, glyphosate-based herbicides also impair the glomerular part of the kidneys, inducing fragmented glomerulus and mitochondria necrosis [19,20]. Thus, mitochondrial alterations might be a key mechanism underlying glyphosate-based herbicides’ deleterious effects on the kidneys.

The aim of this study was therefore to investigate, for the first time, whether glyphosate alone, or associated with surfactants in the very common Roundup^®^ formulation, impairs kidneys’ mitochondrial respiration and production of oxidative stress both at the cortex and medulla levels.

Hypothesizing that tubular cells are more prone to damage in view of their energy needs, we also examined these potential deleterious effects in cultured tubular HK2 cells, considering that Roundup^®^ may induce genetic damage and cause alterations in the DNA repair system in human embryonic kidney cells [21].

## 2. Results

### 2.1. More than Glyphosate Alone, Roundup^®^ Significantly Impairs Kidneys’ Mitochondrial Respiration

#### 2.1.1. Renal Cortex Mitochondrial Respiration

**Figure 1:**
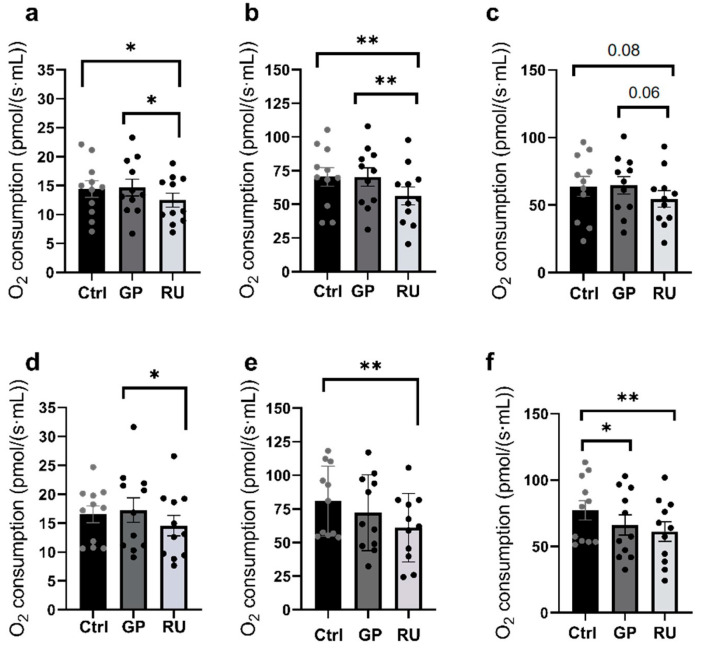
Effect of glyphosate and Roundup^®^ on mitochondria isolated from renal cortex (**a**–**c**) or medulla (**d**–**f**) following incubation of GP or RU for 30 min at 500 µM at 37 °C. (**a**,**d**) Oxygen consumption rate after addition of glutamate/malate and ADP substrates (OXPHOS CI) (n = 11 for each group). (**b**,**e**) Oxygen consumption rate after addition of glutamate/malate/ADP and succinate substrates (OXPHOS CI + II) (n = 11 for each group). (**c**,**f**) Oxygen consumption rate after titration by FCCP (ETS CI + II) (n = 11 for each group). * *p* < 0.05; ** *p* < 0.01.

For the renal cortex, mitochondrial respiration in the OXPHOS CI state was significantly decreased in the Roundup^®^ (RU) group compared to the Ctrl group (−13.5%, *p* = 0.04) and the glyphosate (GP) group (−15%, *p* = 0.02) (14.40 ± 1.42, 14.66 ± 1.46, and 12.46 ± 1.22 pmol/(s·mL) for the control, GP, and RU groups, respectively) (Figure 1a).

During the OXPHOS CI + CII state, oxygen consumption was significantly decreased in the RU group compared to the Ctrl group (−20.1%, *p* = 0.009) and the GP group (−20%, *p* = 0.008) (70.39 ± 6.81 and 56.23 ± 6.73 pmol/(s·mL), respectively) (Figure 1b).

In the ETS CI + II state, a non-significant decrease in O_2_ consumption was observed in the RU group compared to the Ctrl group (−14.7%, *p* = 0.08) and the GP group (−15.6%, *p* = 0.06) (Figure 1c).

#### 2.1.2. Renal Medulla Mitochondrial Respiration

Regarding the renal medulla, Figure 1d shows a significant decrease in mitochondrial respiration by OXPHOS CI in the RU (500 µM) exposed group compared to the group exposed to glyphosate (−15.7%, *p* = 0.03). However, there is no significant decrease in oxygen consumption between the control group and the RU exposed group (16.53 ± 1.46, 17.25 ± 2.13, and 14.54 ± 1.76 pmol/(s·mL) for the control, GP, and RU groups, respectively).

Figure 1e illustrates the OXPHOS CI + CII status of mitochondrial respiration and highlights a significant decrease in oxygen consumption (−24.5%, 80.82 ± 7.88, and 61.03 ± 7.67 pmol/(s·mL), respectively; *p* = 0.003) in the RU exposed group compared to the control. The GP exposed group shows no significant variation. 

Finally, Figure 1f, illustrating the ETS CI + II condition, shows that there is a significant decrease in oxygen consumption in the GP group compared to the Ctrl group (−14.2%, *p* = 0.02). We also observe a significant decrease in mitochondrial respiratory capacity in the RU group compared to the Ctrl group (−20.7%, *p* = 0.001) (77.17 ± 7.31, 66.22 ± 7.63, and 61.18 ± 7.33 pmol/(s·mL), respectively).

#### 2.1.3. Comparison Between Cortex and Medulla Mitochondrial Respiration

**Figure 2:**
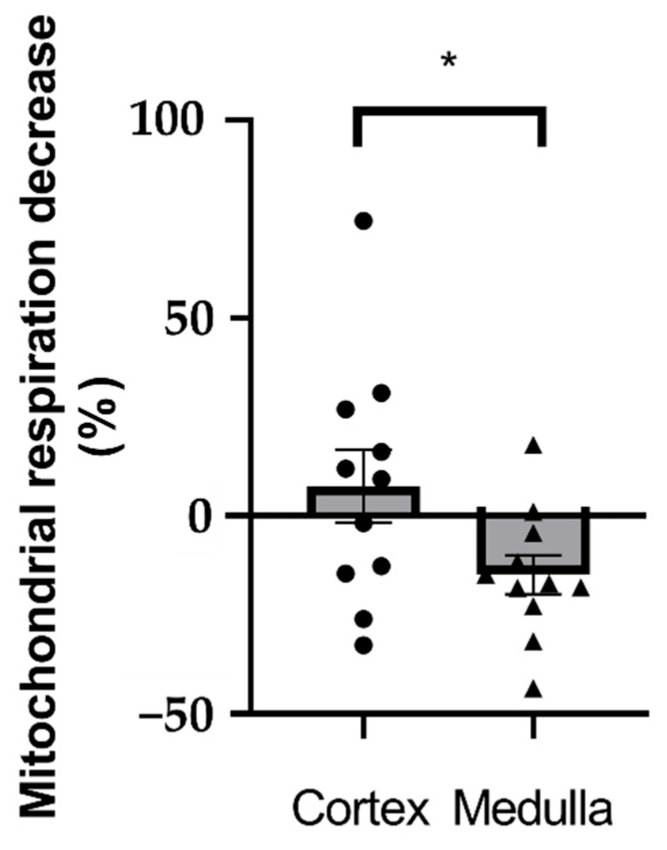
A comparison of the effect of glyphosate on mitochondria isolated from the renal cortex vs. the renal medulla. Oxygen consumption in percent of control (n = 11 for each group). * *p* < 0.05. GP: glyphosate.

When considering maximal mitochondrial respiration after FCCP, there is a significant difference in the effects of GP. Thus, GP significantly decreased renal medulla mitochondrial respiration compared to the renal cortex (7.47 ± 9.17 vs. −14.86 ± 4.91%, *p* = 0.04; Figure 2).

#### 2.1.4. HK2 Cells Mitochondrial Respiration

To study the longer-term exposure of GP and RU, we investigated a human tubular renal cell line model using the HK2 cells exposed to either glyphosate (500 µM) or Roundup^®^ (RU, 500 µM) for 24 h.

**Figure 3:**
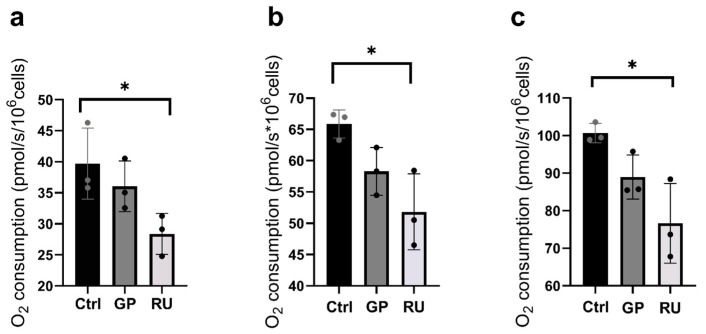
Effect of 24 h exposure to glyphosate and Roundup^®^ on mitochondrial respiration of HK2 cell line following incubation with GP or RU at 500 µM at 37 °C. (**a**) Oxygen consumption rate after addition of glutamate/malate and ADP substrates (OXPHOS CI) (n = 3 for each group). (**b**) Oxygen consumption rate after addition of glutamate/malate/ADP and succinate substrates (OXPHOS CI + II) (n = 3 for each group). (**c**) Oxygen consumption rate after titration with FCCP (ETS CI + II) (n = 3 for each group). * *p* < 0.05. Ctrl: control, GP: glyphosate, RU: Roundup^®^.

Mitochondrial respiration by OXPHOS CI was significantly reduced in the RU-exposed group compared to the control group (−28.5%, 39.71 ± 3.31, and 28.39 ± 1.90 pmol/(s·mL), respectively; *p* = 0.01). In the GP group, no significant variation was observed compared to the control group (Figure 3a).

Figure 3b illustrates the OXPHOS CI + II state of mitochondrial respiration and indicates a decrease in mitochondrial respiration in the RU group compared to the control group (−21.3%, 65.87 ± 1.30, and 51.82 ± 3.50 pmol/(s·mL), respectively; *p* = 0.04). There was a decreasing trend (−11.5%) in the GP group compared to the Ctrl group (Figure 3b).

Finally, under the ETS CI + II condition, there was a significant decrease in oxygen consumption in the RU group compared to the Ctrl group (−23.9%; 100.7 ± 1.48, 76.64 ± 6.13 pmol/(s·mL), *p* = 0.04) and a decreasing trend in the GP group compared to the Ctrl group (−11.6%) (Figure 3c).

### 2.2. Roundup^®^ Enhances Oxidative Stress in Kidneys’ Medulla Mainly at OXPHOS CI + II State

#### 2.2.1. Hydrogen Peroxide Production by Renal Cortex Mitochondria

**Figure 4:**
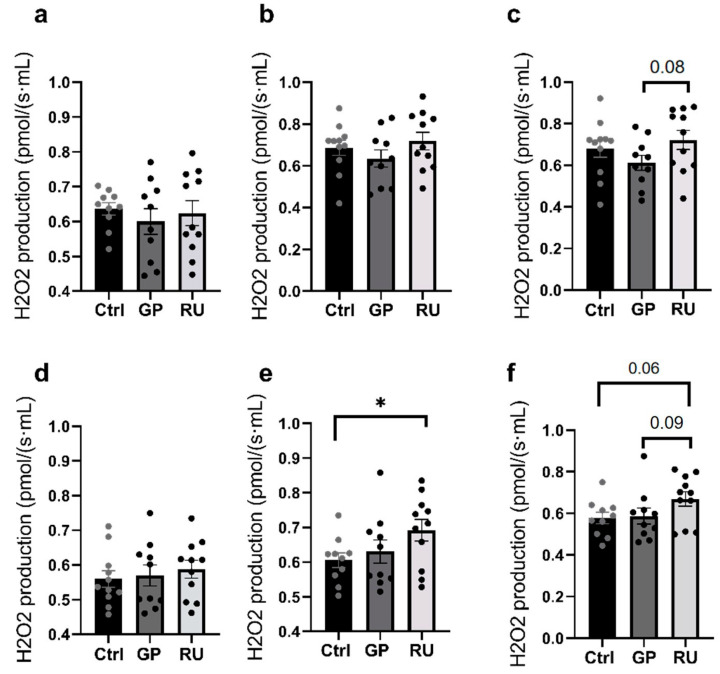
Effect of glyphosate and Roundup^®^ on H_2_O_2_ production in mitochondria isolated from renal cortex (**a**–**c**) or medulla (**d**–**f**) following incubation of GP or RU for 30 min at 500 µM at 37 °C. (**a**,**c**) H_2_O_2_ production rate after addition of glutamate/malate and ADP substrates (OXPHOS CI) (n = 10–11 for each group). (**b**,**e**) H_2_O_2_ production rate after addition of glutamate/malate/ADP and succinate substrates (OXPHOS CI + CII) (n = 10–11 for each group). (**c**,**f**) H_2_O_2_ production rate after FCCP titration (ETS CI + II) (n = 10–11 for each group). * *p* < 0.05.

Although Roundup^®^ tended to increase H_2_O_2_ production in the renal cortex, globally, mitochondrial hydrogen peroxide did not vary significantly between the three groups regardless of the condition (Figure 4a–c).

#### 2.2.2. Hydrogen Peroxide Production by Renal Medulla Mitochondria

Regarding oxygen-derived free radical production, we observed no change when considering OXPHOS CI, and more generally, the GP group did not show any significant difference with the Ctrl group and the RU group regardless of the stage of mitochondrial respiration (Figure 4d).

Nevertheless, the H_2_O_2_ level increased significantly in the RU group compared to the Ctrl group (+14.3%, 0.61 ± 0.02, 0.63 ± 0.03, 0.69 ± 0.03 pmol/(s·mL), *p* = 0.04) when complexes I and II were activated (OXPHOS CI + II) (Figure 4e).

In the ETS CI + II state, there was a non-significant increase in H_2_O_2_ production in the RU group compared to the Ctrl group (+16%, *p* = 0.06) and the GP group (+14%, *p* = 0.09) (Figure 4f). 

#### 2.2.3. HK2 Cells’ Production of Hydrogen Peroxide

**Figure 5:**
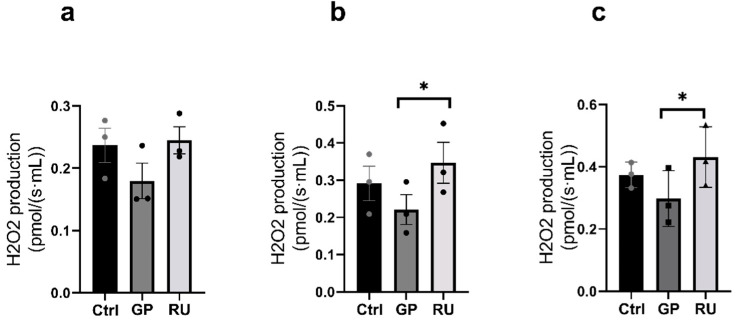
Effect of 24 h exposure to glyphosate and Roundup^®^ on H_2_O_2_ production in HK2 cell line following incubation with GP or RU at 500 µM at 37 °C. (**a**) H_2_O_2_ production rate after addition of glutamate/malate and ADP substrates (CI) (n = 3 for each group). (**b**) H_2_O_2_ production rate after addition of glutamate/malate/ADP and succinate substrates (CI + CI, Vmax) (n = 3 for each group). (**c**) H_2_O_2_ production rate after titration with FCCP (ETS CI + CI) (n = 3 for each group). * *p* < 0.05. Ctrl: control, GP: glyphosate, RU: Roundup^®^.

Regarding reactive oxygen production, no significant difference was observed between the different groups in the OXPHOS CI state (Figure 5a).

However, hydrogen peroxide production was increased in the RU group compared to the GP group (+19%, 0.22 ± 0.04, 0.35 ± 0.05 pmol/(s·mL), *p* = 0.02) during the activation of OXPHOS CI + II respiratory chain complexes (Figure 5b). H_2_O_2_ production was not modified in the GP group compared to the Ctrl group nor between the RU group and the Ctrl group.

Finally, in the ETS CI + II state, H_2_O_2_ production was significantly increased in the RU group compared to the GP group (+23.9%, *p* = 0.04) but was not significantly increased in the RU group compared to the Ctrl group (+11.6%; 0.37 ± 0.02, 0.43 ± 0.06 pmol/(s·mL)) (Figure 5c).

## 3. Discussion

The main results of this study show that, although glyphosate alone decreased maximal uncoupled mitochondrial respiration in the renal medulla, its combination with surfactants was much more toxic to the kidneys. Thus, Roundup^®^ significantly impaired mitochondrial respirations in the renal cortex and medulla and increased hydrogen peroxide production in the medulla when complexes I and II were activated (OXPHOS CI + II).

Similar alterations were observed in tubular HK2 cells after 24 h of exposure to RU. Thus, mitochondrial respiration was significantly impaired in the OXPHOS CI, OXPHOS CI + II, and ETS CI + II states, further supporting the mechanisms (mitochondrial dysfunction and increased reactive oxygen species) involved in renal toxicity induced by GP and Roundup^®^.

### 3.1. Effects of Glyphosate Alone on Kidneys’ Mitochondrial Respiration and Oxidative Stress

Glyphosate alone showed little effect on renal cortex mitochondrial respiration, although it is likely involved in renal toxicity. Our data are similar to other reports, demonstrating fewer deleterious effects of GP alone compared to formulations containing surfactants [22].

Nevertheless, glyphosate significantly impaired maximal uncoupled mitochondrial respiration in the renal medulla. This suggests enhanced deleterious effects when mitochondria function at their maximal capacities. Accordingly, the tubular part of the kidney might be more sensitive to glyphosate toxicity, considering the higher need in energy and therefore the enhanced number of mitochondria in tubular segments as compared to glomeruli mainly localized in the renal cortex. Accordingly, although not reaching statistical significance, mitochondrial respiration tended to be impaired in HK cells, considered tubular cells.

Such a difference might be explained by the fact that the membrane potential of the medulla is lower than that of the cortex [23]. This difference in membrane potential might be due to cardiolipin, a lipid exclusive to mitochondria, representing 18% of the molecules of the inner membrane and responsible for its high impermeability to protons. There is less cardiolipin in the renal medulla compared to the renal cortex [24]. Additionally, Astiz et al. highlighted that glyphosate decreased the amount of cardiolipin through peroxidization, subsequently leading to a decrease in membrane potential [25]. Furthermore, herbicides provoked vacuole formation within the renal tubules together with impaired bioenergetic function in zebrafish embryos [26].

### 3.2. Roundup^®^ Decreased both Renal Cortex and Medulla Mitochondrial Respiration and Impaired Tubular Cell Mitochondria with Enhanced Hydrogen Peroxide Production

#### 3.2.1. Effects of Roundup^®^ on Renal Cortex and Medulla

At the cortical level, Roundup^®^ induced a significant decrease in mitochondrial respiration in the CI and CI + II states compared to the control mitochondria. At the medullary level, Roundup^®^ reduced mitochondrial respiration in the OXPHOS CI, CI + II, and maximal respiration (ETS) states. 

Thus, overall, Roundup^®^ was more toxic than glyphosate alone to the renal cortex and medulla mitochondria. Previous reports support that POEA modifies the toxicodynamics of glyphosate, promoting its entry into the cell and thus increasing its toxic effects. Our results are consistent with Peixoto’s studies on isolated liver mitochondria, highlighting that, unlike Roundup^®^, glyphosate alone has little effect on mitochondria [22]. 

Furthermore, increased hydrogen peroxide production in the renal medulla after Roundup^®^ exposure when investigating mitochondrial complex II might also contribute to tubular alterations.

#### 3.2.2. Effects of Roundup^®^ on HK2 Cell Line

Given the potentially higher sensitivity of the renal medulla containing mainly tubules, the study of mitochondria-rich renal cells from the proximal tubule was of interest. After 24 h of exposure, Roundup^®^ inhibited mitochondrial respiration in the CI, CI + II, and ETS states. This was associated with increased oxidative stress with elevated H_2_O_2_ production. Proximal tubule cells actively reabsorb substances, including sodium, potassium, calcium, phosphate, glucose, amino acids, and water. They use active transporters that require energy, particularly ATP, and mitochondrial dysfunction impacts tubular reabsorption [27]. 

The clinical relevance of these abnormalities was not investigated in our study, but links between renal mitochondrial dysfunction, oxidative stress, and chronic or acute kidney failure have been reported. Impaired mitochondrial respiratory mechanism has been described in patients with chronic kidney disease, associated with increased oxidative stress [28]. In addition, reduced mitochondrial respiratory chain complex I activity was implicated in acute kidney injury [29]. 

This is consistent with our findings, and mitochondrial dysfunction is a recognized pathogenic element of acute kidney injury and a cause of tubular cell dysfunction and death. Renal injury is associated with increased production of reactive oxygen species in mitochondria with decreased ATP production, and cytochrome c release was also associated with epithelial cell and kidney damage. In addition, the surfactant has a synergistic effect with glyphosate, triggering apoptotic and necrosis processes in H9C2, human intestinal epithelial Caco-2, and hepatocyte HepG2 cell lines [30,31,32,33,34,35].

## 4. Material and Methods

### 4.1. Study Design

This research was conducted on mitochondria isolated from rats’ kidneys and on cultured cells.

#### 4.1.1. Experimental Design

**Figure 6:**
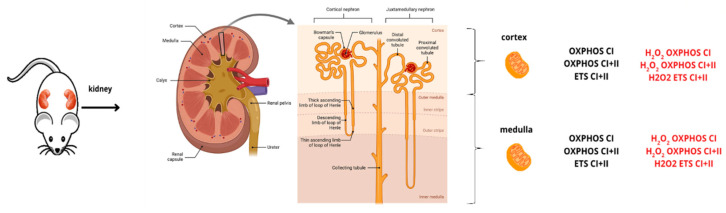
Experimental design.

Eleven 5-month-old male Wistar rats from the Janvier Lab (Janvier, Le Genest-St-Isle, France) were studied. The animals were kept in an enriched environment at 22 ± 2 °C under a 12 h light–dark cycle with free access to water and food. Anesthesia was induced and maintained by the inhalation of isoflurane (4%) and oxygen in an induction chamber. Animals were euthanized before tissue collection. The left kidney was systematically removed, and the separation of the cortex and medulla was performed (Figure 6). Then, the tissues were placed in cold isolation buffer (sucrose [250 mM], Tris/HCl [10 mM], EGTA [0.1 mM], pH 7.4) at 4 °C.

The experiment was conducted in accordance with the principles of laboratory animal care and the European Union Guidelines (86/609/EU) and the regulations of the Committee for the Care and Use of Laboratory Animals (Cremeas, France, decree 2020-274, article R. 214-89), as previously reported [36].

After euthanasia, the left kidney was systematically collected and dissected under a binocular microscope to separate the cortex and the medulla. Then, mitochondria from each part were extracted by differential centrifugation. Mitochondrial function was studied using Oroboros-2K, specifically O_2_ consumption and H_2_O_2_ production. OXPHOS CI was obtained after the injection of glutamate (5 mM), malate (2 mM), and ADP (2 mM). OXPHOS CI + II was then obtained by adding succinate (10 mM). Finally, to determine the maximal respiratory capacity (ETS CI + II), FCCP (0.25 µM) was injected. The image was created on BioRender.com

#### 4.1.2. Cell Culture

Human kidney-2 cells (HK-2, Kidney Proximal Tubule; Human from American Type Culture Collection) were grown in Dulbecco’s modified Eagle’s medium supplemented with 10% fetal bovine serum at 37 °C under a humidified atmosphere with 5% CO_2_. 

### 4.2. Solutions Used

Glyphosate [N-(phosphomethyl)-glycine] (100% purity, Pestanal^®^) was prepared at an initial concentration of 12 g/L. Roundup^®^ was prepared to contain 12 g/L of glyphosate (stock concentration: 360 g/L). Isolated mitochondria and cells were exposed to a final concentration of 500 μM (85 mg/L) of glyphosate alone or in Roundup^®^. This concentration is consistent with previous investigations at the cellular level and with the values observed in human blood after exposure to herbicide. Indeed, the average blood concentration of glyphosate was 61 mg/L (range 0.6–150 mg/L) in mild and moderate intoxications and varied from 690 to 7480 mg/L in cases of fatal issues [37,38]. 

### 4.3. Mitochondrial Extraction

Mitochondria were isolated by sequential centrifugation as previously reported [39]. After washing, the mitochondrial suspension was homogenized using a dissociator (Miltenyi Biotec, Bergisch Gladbach, Germany). The homogenate was first centrifuged at 3000 rpm for 3 min at 4 °C, and then the resulting supernatant was centrifuged at 8000 rpm for 10 min at 4 °C. Mitochondria were then washed and concentrated by centrifugation at 11,000 rpm for 5 min at 4 °C. The mitochondrial pellet was resuspended in an ice-cold buffer (50 mM Tris, 70 mM sucrose, and 210 mM mannitol; pH 7.4 at +4 °C), and the protein concentration was measured by Bradford assay.

The supernatant was then centrifuged at 4306 g for 10 min at 4 °C, and the resulting pellet was resuspended in isolation buffer. A final centrifugation at 4306 g for 10 min at 4 °C was performed, after which the pellet was resuspended in suspension buffer (sucrose [250 mM], Tris/HCl [10 mM], pH 7.4). These last two centrifugations allowed for the pelleting and washing of mitochondria.

### 4.4. Mitochondrial Respiration

#### 4.4.1. Isolated Mitochondria

Renal mitochondrial respiration was determined at 37 °C under continuous stirring using a high-resolution oxygraph (Oxygraph-2K, Oroboros instruments, Innsbruck, Austria) containing two Clark-type electrodes. An amount of 0.8 mg of mitochondria was placed in 2 mL of respiration buffer (Miro5 + creatine: EGTA 0.5 mM, MgCl2 3 mM, K lactobionate 60 mM, Taurine 20 mM, KH2PO4 10 mM, HEPES 20 mM, sucrose 110 mM, BSA 1 mg/mL, creatine 20 mM).

Oxygen consumption, mainly related to the mitochondria introduced into the respiratory chambers, was then measured. Mitochondrial respiration was determined using sequential injection of specific substrates and inhibitors of the electron transport chain. The initial substrates glutamate (5 mM), malate (2 mM), and ADP (2 mM) were used to stimulate the activity of complexes I, III, IV, and V, namely the OXPHOS CI state. Subsequently, the addition of succinate (10 mM) led to the activation of complex II in addition to the already active complexes, namely the OXPHOS CI + II state. Finally, we measured the maximal respiratory capacity (ETS CI + II). This respiratory state was assessed by stepwise titration of an uncoupling agent such as carbonyl cyanide-p-trifluoromethoxyphenylhydrazone (FCCP 0.25 µM).

Data were expressed in pmol/(s·mL).

#### 4.4.2. HK-2 Cells

The same protocol was used for cells whose membranes were permeabilized using saponin (0.125 mg/mL) at the start of the protocol. Data were expressed as pmol/(s·10^6^ cells).

### 4.5. Mitochondrial H_2_O_2_ Production

Hydrogen peroxide (H_2_O_2_) production was measured using the high-resolution respirometer (Oxygraph-2k; Oroboros Instruments, Innsbruck, Austria) simultaneously with mitochondrial respiration in isolated mitochondria and HK2 cells.

H_2_O_2_ production was detected using Amplex Red (Invitrogen, Waltham, MA, USA), which reacts in a 1:1 stoichiometry with H_2_O_2_ in a reaction catalyzed by horseradish peroxidase (HRP; Sigma Aldrich, St. Louis, MO, USA), producing the fluorescent compound resorufin, as previously reported [40].

Resorufin has excitation/emission wavelengths of 563/587 nm and remains very stable once formed. Data were expressed as pmol H_2_O_2_/s/10^6^ cells for cells and as pmol H_2_O_2_/s/mL for isolated mitochondria.

### 4.6. Statistical Analysis

Statistical analyses were performed using Prism software (GraphPadPrism 8.4.3, GraphPad Software, San Diego, CA, USA). Results are expressed as mean ± SEM (Standard Error of the Mean), with “n” representing the number of samples. Normality of data distribution was assessed using the Shapiro–Wilk test.

Depending on the conditions, comparisons between the control, glyphosate, and Roundup^®^ groups were conducted using an ANOVA test. Fisher’s LSD post hoc test was used for group comparisons. If the data did not follow a normal distribution, a non-parametric Friedmann test was performed. For the comparison of two groups following a normal distribution, Student’s *t*-test was used. The significance threshold for the *p*-value was set at 0.05.

## 5. Conclusions

This study demonstrates that RU has greater toxic effects than GP on renal filtration and reabsorption structures, with the medulla likely being more prone to injury than the cortex. The underlying mechanisms involve impaired mitochondrial respiration and oxidative stress, which warrants approaches aimed at improving mitochondrial function and reducing oxidative stress to prevent or reduce the deleterious effects of these herbicides, which are used worldwide, on the kidneys. Furthermore, a reduction in the use of GP and RU appears essential, along with increased caution to better protect users and the general population.

## Data Availability

The original contributions presented in this study are included in the article. Further inquiries can be directed to the corresponding author.
